# Guided bone regeneration in staged vertical and horizontal bone augmentation using platelet-rich fibrin associated with bone grafts: a retrospective clinical study

**DOI:** 10.1186/s40729-020-00266-y

**Published:** 2020-10-17

**Authors:** Carlos Alberto Amaral Valladão, Mabelle Freitas Monteiro, Julio Cesar Joly

**Affiliations:** 1 Department of Implantology and Periodontology, São Leopoldo Mandic Dentistry Research Center, R. Dr. José Rocha Junqueira, 13 - Pte. Preta, Campinas, SP 13045-755 Brazil; 2 Department of Prosthodontics and Periodontics, Piracicaba Dental School, University of Campinas, Piracicaba, SP Brazil

**Keywords:** Guided bone regeneration, Alveolar bone grafting, i-PRF, Dental implants, Alveolar ridge augmentation, Platelet-rich fibrin

## Abstract

**Background:**

The use of guided bone regeneration (GBR) for vertical and horizontal bone gain is a predictable approach to correct the bone defects before implant installation; however, the use of different protocols is associated with different clinical results. It is suggested that platelet-rich fibrin (PRF) could improve the outcomes of regenerative procedures. Thus, this study aimed to describe the bone gain associated with GBR procedures combining membranes, bone grafts, and PRF for vertical and horizontal bone augmentation.

**Materials and methods:**

Eighteen patients who needed vertical or horizontal bone regeneration before installing dental implants were included in the study. The horizontal bone defects were treated with a GBR protocol that includes the use of a mixture of particulate autogenous and xenogenous grafts in the proportion of 1:1, injectable form of PRF (i-PRF) to agglutinate the graft, an absorbable collagen membrane covering the regenerated region, and leukocyte PRF (L-PRF) membrane covering the GBR membrane. The vertical bone defects were treated with the same grafted mixture protected by a titanium-reinforced non-resorbable high-density polytetrafluoroethylene (d-PTFE-Ti) membrane and covered by L-PRF. The bone gain was measured using a cone-beam computed tomography at baseline and after a period of 7.5 (± 1.0) months.

**Results:**

All patients underwent surgery to install implants after this regenerative protocol. The GBR produces an increase in bone thickness (*p* < 0.001) and height (*p* < 0.005) after treatment, with a bone gain of 5.9 ± 2.4 for horizontal defects and 5.6 ± 2.6 for vertical defects. In horizontal defects, the gain was higher in the maxilla than in mandible (*p* = 0.014) and in anterior than the posterior region (*p* = 0.033). No differences related to GBR location were observed in vertical defects (*p* > 0.05).

**Conclusion:**

GBR associated with a mixture of particulate autogenous and xenogenous grafts and i-PRF is effective for vertical and horizontal bone augmentation in maxillary and mandibular regions, permitting sufficient bone gain to future implant placement.

**Trial registration:**

REBEC, RBR-3CSG3J. Date of registration—19 July 2019, retrospectively registered. http://www.ensaiosclinicos.gov.br/rg/RBR-3csg3j/

## Background

The volumetric alteration in the maxillary and mandibular bone is a critical consequence of tooth loss, limiting the rehabilitation with dental implants [1], and the reduction in volume is described between 29 and 63% horizontally and between 11 and 22% vertically after 6 months of tooth loss [2]. Many techniques and biomaterials have been proposed to correct these discrepancies. For horizontal gain, autogenous and allogeneic block grafts; autogenous, xenogenous, and alloplastic particulate grafts; expansion of the alveolar crest; and guided bone regeneration (GBR) have been proposed [1, 3]. As an alternative for vertical loss, short implants (smaller than 7 mm), lateralization of the lower alveolar nerve, autogenous block graft, osteogenic distraction, use of growth factors and tissue engineering, and GBR were described [4, 5].

Among those techniques, the use of particulate grafts and GBR has the benefit of reducing the morbidity [6, 7], unpredictability [8], and complications [9] related to surgical techniques. The autogenous bone grafts presented osteogenic, osteoinductive, and osteoconductive properties [10]. However, they are usually removed from intraoral or extraoral areas, requiring a second surgical site, and consequently, increasing the morbidity [6, 7]. Homogeneous, heterogeneous, and synthetic grafts, on the other hand, only present the property of osteoconduction. However, when mixed with autogenous bone or growth factors, they serve as a framework favoring the adhesion and proliferation of osteoprogenitor cells [10].

GBR also promotes alveolar bone gain with predictable and stable results [11]. The basic principle of GBR involves the use of a mechanical barrier that will isolate the surgical site from epithelial and connective tissue cells, allowing the proliferation of osteogenic cells and bone formation [12]. Usually, the membrane is used over the grafted material once the bone particles can favor the space maintenance and stability of the fibrin clot formed on the surgical site, two principles of GBR [13]. A more recent study proposes that the membrane acts as a bioactive compartment that facilitates cells’ attraction that release signals and growth factors for remodeling, regeneration, and vascularization (BMP-2, FGF-2, TGF-β, VEGF) [14]. Membranes are classified as resorbable or non-resorbable membranes, according to the type of material used: synthetic (derived from polymers) or of animal origin [15]. The most widely used absorbable membranes are obtained from type I collagen or from a combination of types I and III collagens, and the sources of this collagen include bovine tendon, bovine dermis, sheepskin, or swine dermis [16]. The most used non-absorbable membranes are those of high-density polytetrafluoroethylene (d-PTFE) with or without titanium reinforcement. These membranes provide an effective barrier function, favor space maintenance, and are biocompatible. However, they need to be removed, creating the disadvantage of a second surgical procedure.

Recently, the association of platelet-rich fibrin (PRF) to regenerative procedures has been proposed. The biological plausibility of PRF is related to the increasing concentration of growth factors and other molecules related to angiogenesis, stem cell migration, and the osteogenic differentiation on the regenerated site, improving the biological capabilities, tissue formation, and healing [17, 18]. However, there is a lack of evidence related to their use in hard-tissue formation [17]. Thus, this retrospective study aimed to describe the bone gain associated with GBR protocols combining bone grafts, PRF, and membranes for vertical and horizontal bone augmentation.

## Methods

This retrospective study included patients who came to a private clinic in Brasília, DF, Brazil, from March 2013 to July 2019. The study was approved by the ethics and research committee of São Leopoldo Mandic School of Dentistry (Campinas, Brazil) number 10586419.0.0000.5374, and it was registered at the Brazilian Clinical Trials Registry (REBEC) under the number RBR-3csg3j. After a health history evaluation and anamnesis, the patients were clinically evaluated, and complementary exams of cone-beam computed tomography (CBCT) were performed. Were included in the study patients who needed vertical or horizontal bone regeneration before installation of dental implants in a partially edentulous alveolar ridge, with good physical health and able to maintain good oral hygiene. The horizontal bone deficiencies included in the study were classified according to Cawood and Howell [19] as class IV (knife-edge ridge form, adequate in height and inadequate in width), while the vertical atrophies were classified as class V (flat ridge form, inadequate in height and width). All patients should present a tomographic exam before GBR. This study’s exclusion criteria were as follows: patients who were smokers, alcoholics, use illicit drugs, diabetics, and patients with uncontrolled systemic diseases. A total of 23 patients matched the inclusion criteria, from which five (three patients had diabetes, one was a current smoker, one used illicit drug) were removed from the analysis based on the exclusion criteria.

### CBCT exams and bone measurements

The image of the patient’s maxillary and mandibular arches was acquired by the i-CAT Next Generation Model CT scanner (Imaging Sciences International LLC, Hatfield, PA, USA). The image parameters were adjusted to 120 kVp, 37.07 mAs, scan time 26.9 s, resolution 0.25 mm, and a field of view (FOV) that varied based on the digitized region. Existing bone defects were measured in the tomographic sections obtained. The measurements were taken at the site corresponding to the missing tooth (or missing teeth), directly on the printed cuts, provided by the radiological clinic and confirmed, by digital measurement, through the analysis of the DICOM files (Digital Imaging and Communications in Medicine), with specific software (Nobel Clinician, Nobel Biocare). Measurements were taken before bone graft surgery and after 7.5 (± 1.0) months. The same calibrated examiner CV performed all measurements.

**Fig. 1:**
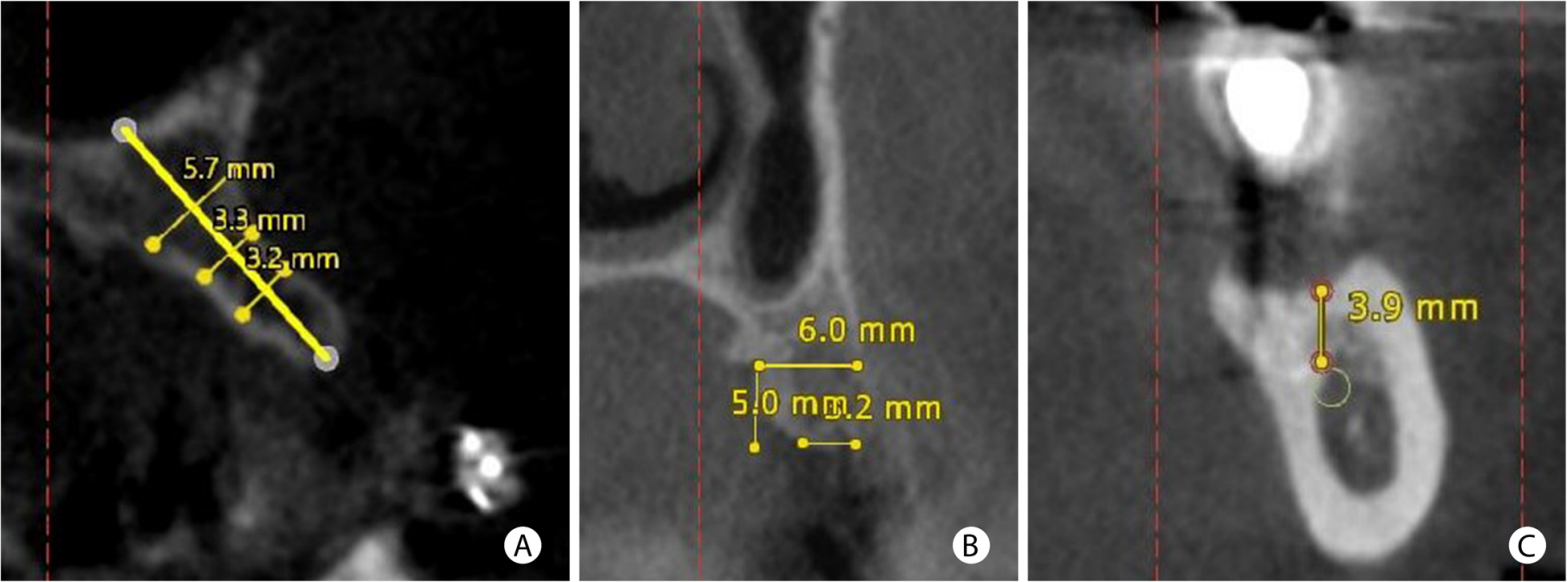
The methodology used to measure bone defects in the CBCT. **a** For horizontal bone defects, the tomographic measurement was performed at three different points, and three horizontal lines recorded bone thickness at 5, 7, and 11 mm from the alveolar crest. **b** In regions close to the maxillary sinus with a horizontal deficiency in the remaining bone crest, the measurement was performed 5 mm from the bone crest. **c** Vertical bone defect measured from the viable edge of the alveolar crest to the lower alveolar nerve

Figure 1 describes the methodology used to standardize the tomographic measurements. The existing residual bone thickness was measured at three different points for horizontal defects, following a previously described methodology [20]. A perpendicular line was drawn following the inclination of the residual bone. Subsequently, three horizontal lines registered bone width at 5, 7, and 11 mm from the crest. The average value of the three measurements was recorded. In cases of horizontal increases in posterior regions of the maxilla concomitant with vertical bone augmentation and maxillary sinus lifting, only one measurement was performed: 5 mm from the edge of the remaining bone crest. For vertical defects, the baseline measures were performed from the viable edge of the alveolar crest up to the limit of anatomical structures limiting the treatment (for mandible: inferior alveolar nerve, incisor nerve, mental foramen, and basal cortical limit; for maxilla: the base of the nasal floor and the maxillary sinus). After the graft-healing period, a new CBCT exam was performed and evaluated in the same region of the first measurement. The final values were subtracted from the baseline measurements to obtain the bone gain’s extent after GBR. The error in the radiographic evaluation was determined using repeated measures in five randomly selected patients. The mean difference was 0.5 ± 0.25 mm between examinations.

### Surgical protocol for GBR treatment

All surgeries were performed, in a private clinic, by the same professional CV. The patients were premedicated with amoxicillin 2 g, 1 h before the surgical procedure. For allergic patients, the antibiotic of choice was clindamycin 600 mg, 1 h before the surgery. Patients rinsed a 0.12% chlorhexidine solution. All surgeries were performed under intravenous sedation performed by an anesthesiologist.

After local anesthetic infiltration with 4% articaine 1:100,000 (Nova DFL, Brazil), a total mucosal periosteum incision was made in keratinized gingiva in the edentulous alveolar crest, and this incision continued intrasulcularly, involving at least two teeth mesially and distally (when present) in addition to the edentulous region, both lingually and buccally. Two relaxing vertical incisions were made in the buccal face, mesially and distally to the defect. In free extremity regions, vertical incisions were made about 5 mm beyond the treated surgical site. After the initial incision, a total mucous-periosteum flap was obtained using periosteal elevators. After elevation of the flap, perforations were performed in the recipient area’s cortical bone with a 1.5 to 2-mm spiral drill. The purpose of these perforations was to facilitate angiogenesis and neovascularization and to provide osteoprogenitor cells for the grafted region [21, 22]. The particulate autogenous bone was collected using bone scrapers (Safescraper® TWIST or Micross, Meta, Italy) or a trephine drill. The donor regions were the sites surrounding the defect, external oblique line, or mandibular retromolar areas. The collected particulate autogenous bone was mixed with ABBM in equal proportions producing a composite bone graft.

**Fig. 2:**
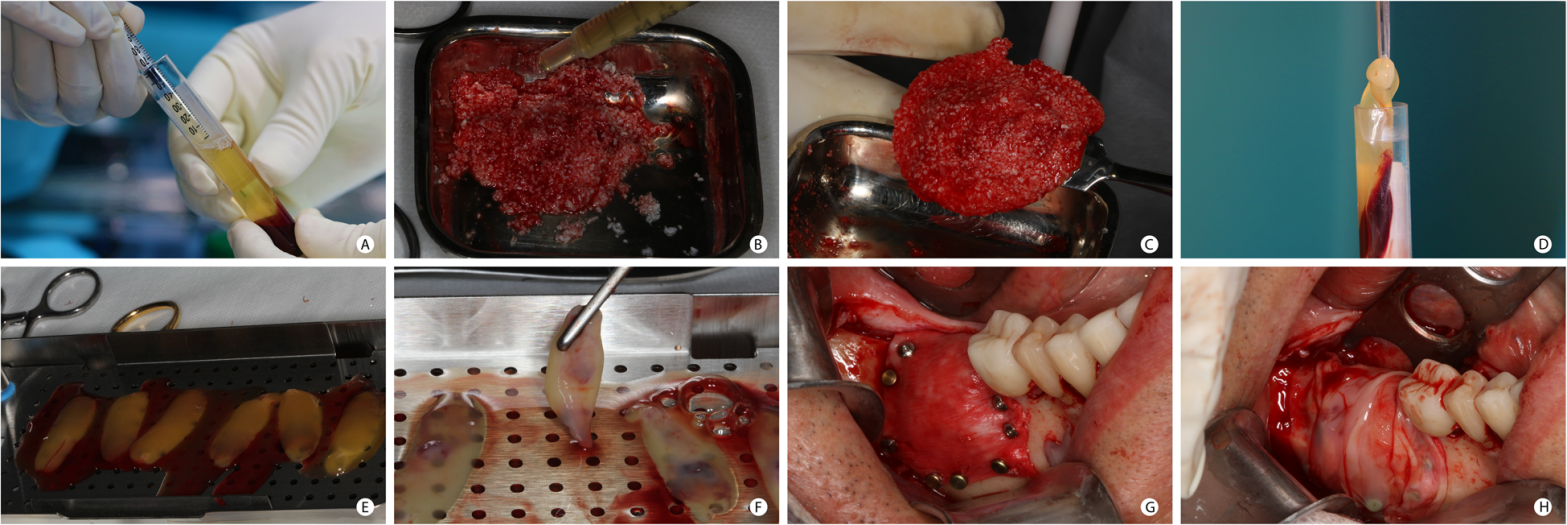
PRF preparation and usage in the GBR procedure. **a** - i-PRF, obtained after centrifugation of the tube containing the patient’s blood at 2700 rpm/400 g RCF for 3 min (IntraSpin ™—Intra-Lock), was aspirated with a plastic syringe from the tube. **b** Agglutination of the graft particles (autogenous and xenogenous graft) with i-PRF. **c** Bone graft after agglutination with i-PRF. Note the consistency of the graft mixture and its easy manipulation. **d** L-PRF clot, obtained after the blood centrifugation for 12 min, was removed from the tube and separated from the red blood cells. **e** L-PRF clots. **f** Preparation of L-PRF membranes with compression in a metal box (Xpression ™ Box—Intra-Lock). **g** Clinical view of the GBR site immediately before the use of L-PRF membranes. **h** L-PRF membranes covering the GBR region before suture

During the surgical procedure, the patient’s blood was collected with venipuncture into centrifugation tubes to produce the blood derivatives. The injectable form of PRF (i-PRF) was used to agglutinate the bone graft particles, and the leukocyte platelet-rich fibrin (L-PRF) was used to produce a biomembrane to cover the GBR membranes. i-PRF was obtained after centrifugation of 2 non-ridged tubes without any additive [23] (Vacuette® tube Z No Additive, Greiner Bio-One International GmbH, Austria), containing 8 ml of the patients’ blood for 3 min, at a speed of 2700 rpm, in a centrifuge (Intra-Lock International Inc, USA) [24]. The L-PRF was obtained after centrifugation of 6 plastic serum tubes, with silica clot activator, silicone-coated interior (BD Vacutainer® Serum Tubes, Becton, Dickinson and Company, USA) containing 8 ml of the patients’ blood for 12 min, at a speed of 2700 rpm. A total of 8 tubes were collected from each patient, regardless of the bone defect’s extent. The usage of i-PRF and L-PRF is described in Fig. 2. Figure 2 a–c describe the agglutination of graft particles with i-PRF, acquiring a form and consistency that facilitates the graft positioning in the surgical site. Figure 2 d–h describe the production and clinical use of L-PRF, positioned over the collagen or d-PTFE membranes before the suture.

**Fig. 3:**
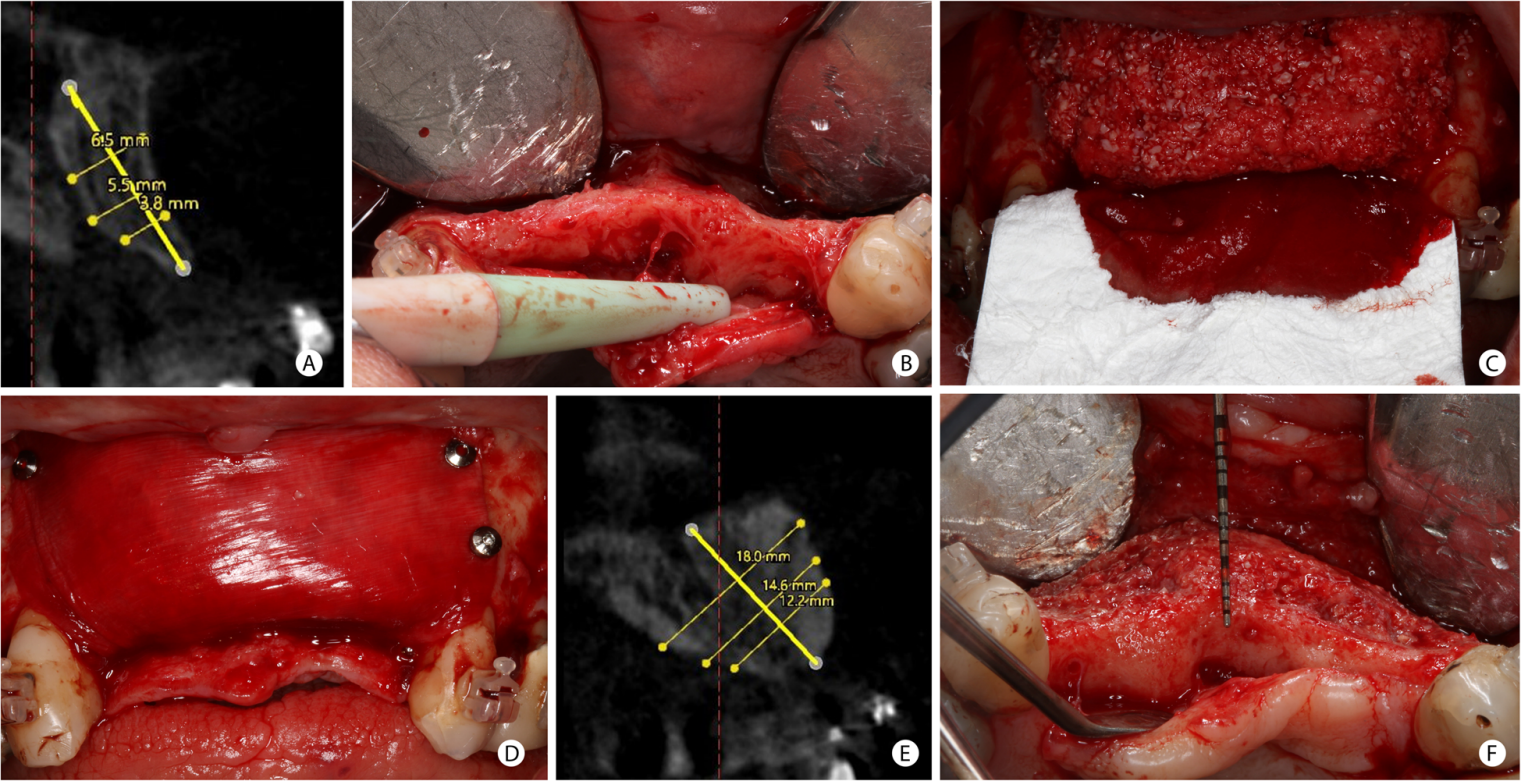
GBR procedure for horizontal bone increase in the anterior maxilla. **a** Tomographic measurement of the horizontal defect in the upper right central incisor region. **b** Clinical aspect of the horizontal defect in the anterior maxilla. **c** Mixture of particulate autogenous and ABBM grafts (in the proportion 1:1) agglutinated with i-PRF positioned in the horizontal defect and the initial fixation of the collagen membrane palate. **d** Collagen membrane covering the grafted material and fixed in the residual vestibular bone with thumbtacks. **e** Tomographic bone measure after 8 months of GBR. 9.7 mm of horizontal bone gain was achieved when comparing the baseline and final tomography. **f** Clinical view of the horizontal bone augmentation in the time of implant placement

Patients with horizontal bone defects were treated with the GBR protocol described previously [25], modified using i-PRF to agglutinate the graft. Figure 3 describes this protocol. The bone graft used was a mixture of particulate autogenous bone and bovine inorganic bone graft (Bio-Oss®, GeistlichPharma AG, Wolhusen, Switzerland) in the proportion of 1:1. The mixture was agglutinated with i-PRF to facilitate the graft particles’ aggregation and then positioned in the defect area to be regenerated. As the horizontal bone defects treated in this study had a knife-edge ridge form with adequate height, this bone wall could provide stability for the graft and a non-rigid membrane, such as a collagen membrane. Therefore, the graft mixture was covered with an absorbable xenogenous collagen membrane (double layer of porcine types I and III collagen-Bio-Gide, GeistlichPharma AG, Wolhusen, Switzerland). This type of membrane ensured stabilizing the graft particles and exhibited perfect tissue integration and, thereby, fast vascularization [26]. The membrane was immobilized with thumbtacks (Master Pin Control, Meisinger, Germany). Finally, the L-PRF was positioned over the absorbable membrane before suture.

The selected patients with horizontal bone deficiencies located in the posterior maxilla had a remaining alveolar bone crest with a maximum height of 7 mm up to the maxillary sinus floor and a reduced bone thickness. This morphology is commonly found in the premolar region [27]. These patients were treated with maxillary sinus lifting and simultaneous horizontal GBR procedure for horizontal enlargement of the remaining alveolar bone crest, achieving the appropriate bone height and thickness necessary for the future placement of the implant. The graft material used for sinus lifting was the ABBM (Bio-Oss® Pen, GeistlichPharma AG, Wolhusen, Switzerland) with large particles ranging from 1 to 2 mm [28]. The horizontal GBR treatment followed the same protocol described previously.

**Fig. 4:**
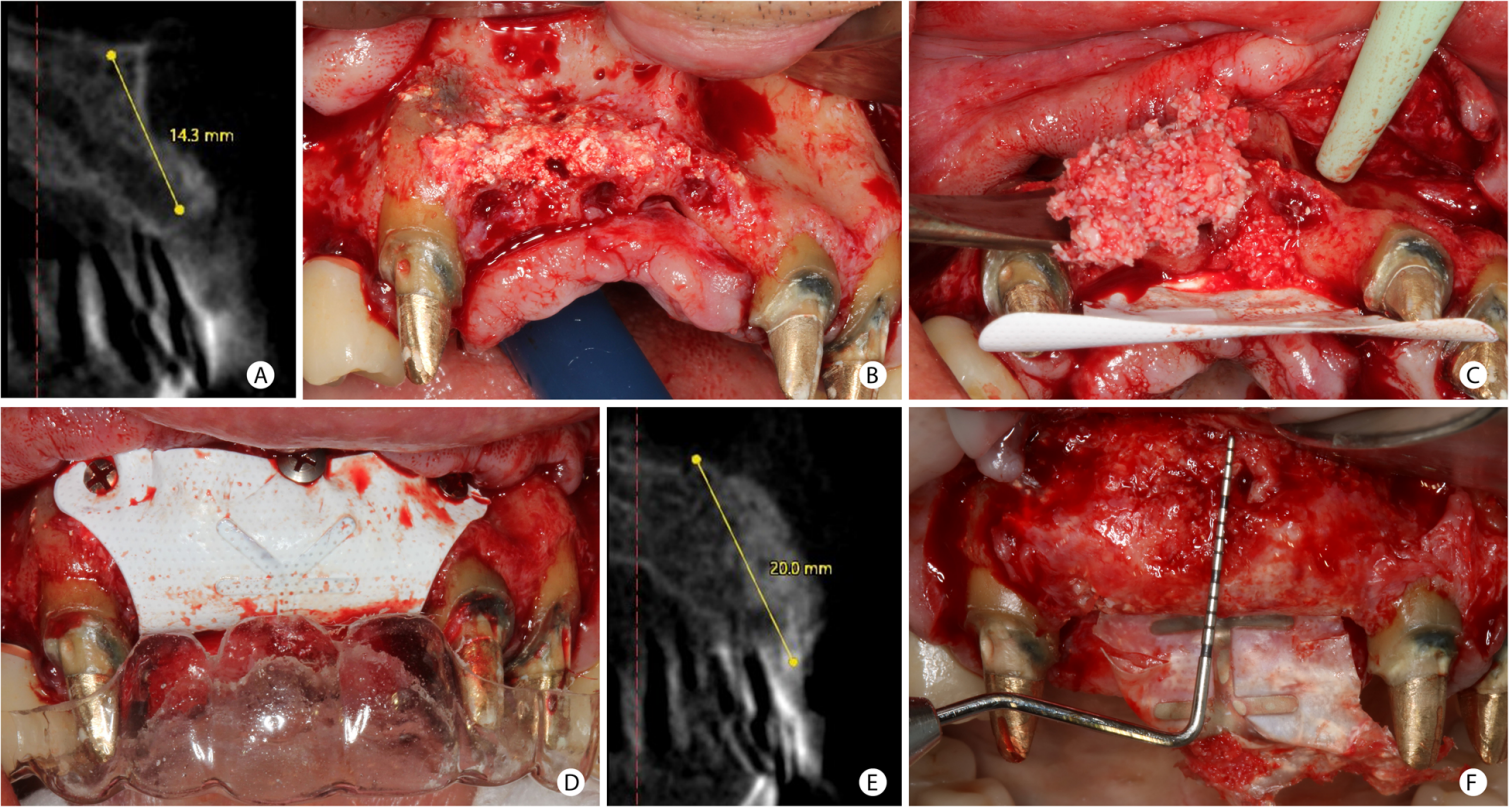
GBR procedure for vertical bone increase in the anterior maxillary region. **a** Tomographic section of the vertical defect in the upper right lateral incisor region. **b** Clinical view of the vertical defect in the anterior maxilla. Observe the alveolar defect after 2 months of removal of poorly positioned implants and the presence of particles from the previous bone graft. **c** d-PTFE-Ti membrane fixed in the palate and the graft mixture being placed in the region. **d** Membrane covering the graft and fixed in the vestibular. **e** CBCT image after 8.5 months. The vertical bone gain of 9.8 mm after comparing the baseline and final CBCT. **f** Clinical aspect of the regenerated region before the implant placement

**Fig. 5:**
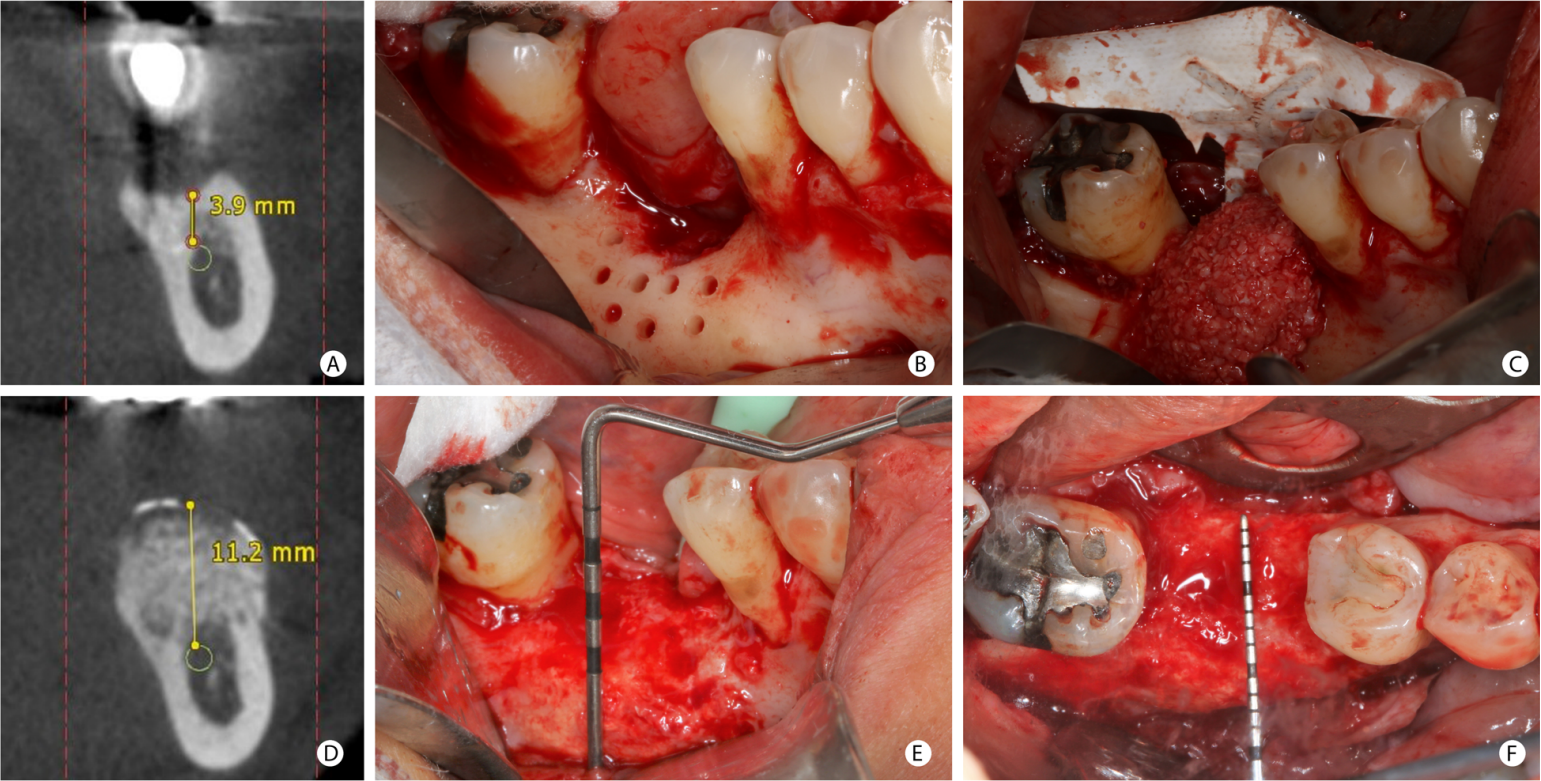
GBR procedure for vertical bone increase in the posterior mandibular region. **a** Tomographic measurement of the vertical bone defect in the position of the mandibular first molar. **b** Clinical aspect of a vertical defect after the cortical bone’s perforations with a spiral drill. **c** Mixture of autogenous and ABBM grafts agglutinated with i-PRF positioned under the d-PTFE-Ti membrane already fixed in the lingual face. **d** The bone height of 11.2 mm measured on CBCT, after 8.5 months. **e** Clinical aspect of the vertical bone augmentation. **f** Occlusal view after GBR

Figures 4 and 5 illustrate the surgical procedure for vertical gain. Patients treated with GBR to correct vertical bone defects received the same graft mixture described for horizontal defects; however, a titanium-reinforced non-resorbable high-density polytetrafluoroethylene (d-PTFE-Ti) membrane (Cytoplast ™ Ti-250, Osteogenics Biomedical, USA) [29] covered the surgical site. This membrane was used since it provides a rigid stabilization of the particulate bone graft, which is essential in vertical bone-augmented sites where there is no remaining bone wall to support the graft. The membrane was fixed with thumbtacks’ aid (Master-Pin-Control Bone System®, Meisinger, Germany) or screws (Pro-fix ™ Precision Fixation System, Osteogenics Biomedical, USA), and L-PRF membranes covered all the regenerated site.

Before the sutures, the flaps were released to obtain a coaptation of the tissues free of tension. A single linear superficial incision in the periosteum region at the bottom of the vestibule was used for vestibular flaps. In mandibular lingual regions, the flap was relaxed using a blunt instrument in the superficial fibers of the mylohyoid muscle, which are tenuously attached to the lingual flap’s periosteum. In the treated edentulous region, the flap was sutured in two layers using horizontal mattress sutures interspersed with simple sutures, while simple sutures were used to close the vertical incisions. The suture was done with non-absorbable PTFE 3-0 thread (Cytoplast™ Suture, Osteogenics Biomedical, USA) removed 15 days after the surgical procedure. As a postoperative medication, the patient used amoxicillin 500 mg of 8/8 h (clindamycin 300 mg 6/6 h, for patients allergic to penicillin) for 10 days, ibuprofen 400 mg 8/8 h for 5 days, and 0.12% chlorhexidine mouthwash, started 24 h after surgery, for 15 days. The patient returned for postoperative evaluation after 1 week, and 15 days for suture removal. The surgical site healed for 6 to 9 months with no clinical intervention, and the patients were evaluated monthly during this period.

### Statistical analysis

Descriptive analyses were carried out to assess the influence of sex, age, and the region of bone defects; the comparison between the initial and final bone thickness and height was made with paired *t* tests. Analysis of variance (ANOVA) for randomized blocks was applied to investigate whether the horizontal and vertical bone augmentation after guided bone regeneration differed regarding the arch and GBR location. Statistical analysis was performed using the SPSS 23 program (SPSS INC., Chicago, IL, USA), with a significance level of 5%.

## Results

Fifty-two regions with bone defects were treated in 18 patients: ten patients had horizontal defects (four males and six females, with a mean age of 59.4 years), and eight patients had vertical defects, with equal proportions between the sexes (mean age 55.4). After completing the GBR treatment, the patients were followed for a period ranging from 1 to 7 years. During the entire postoperative evaluation period, which ranged from the first week to 9 months after the GBR procedure, all clinical cases evolved without complications, such as membrane exposure, wound dehiscence, or infection. New measurements of the grafted regions were obtained through analysis of postoperative tomographic sections obtained after a period of 7.5 (± 1.0) months. All patients underwent surgery to install implants, and at that moment, the bone gain obtained was clinically confirmed. The postoperative bone gain is demonstrated for horizontal defects in Fig. 2 e and f and for vertical defects in Figs. 3 e and f and 4 d and e.

Twenty-nine sites with horizontal bone deficiencies were treated, of which ten (34.5%) were in the upper molar region, five (17.2%) in upper central incisors, four (13.8%) in upper lateral incisors, four (13.8%) in mandibular molars, four (13.8%) in mandibular premolars, one (3.4%) in mandibular canine, and one (3.4%) in mandibular central incisor. Regarding vertical bone defects, 23 sites were treated in the following regions: mandibular molars (34.8%), maxillary central incisor (21.7%), mandibular (17.4%) and maxillary (8.7%) premolars, maxillary lateral incisors (8.7%), maxillary molars (4.3%), and mandibular canines (4.3%).

The measurements of bone thickness, height, and bone increases, performed in the initial and final CBCTs, are described in Table 1. An increase in the bone height and thickness was observed after GBR in vertical (*p* < 0.005) and horizontal (*p* < 0.001) defects, independent of the operated region (arch and location). The horizontal bone increasing was significantly higher in the upper arch than in the mandible (*p* = 0.014) and in the anterior region than in the posterior region (*p* = 0.033). On the other hand, for vertical gain, no difference was observed neither when arches (*p* = 0.451) nor when anterior and posterior regions (*p* = 0.814) were compared.

**Table 1:** Horizontal and vertical bone measures in millimeters (mean ± standard deviation)

		Horizontal			Vertical		
		Baseline	Final	Δ thickness	Baseline	Final	Δ height
General bone measures		5.3 ± 1.7 B	11.3 ± 2.5 A	5.9 ± 2.4	9.0 ± 3.4 B	14.6 ± 4.3 A	5.6 ± 2.6
Arch	Maxilla	5.1 ± 1.4 B	11.5 ± 2.4 A	6.5 ± 2.4 a	11.9 ± 2.3 B	16.9 ± 4.0 A	5.0 ± 2.9 a
	Mandible	6.4 ± 2.3 B	10.2 ± 3.0 A	3.8 ± 1.1 b	7.5 ± 2.9 B	13.4 ± 4.1 A	5.9 ± 2.4 a
Region	Anterior	4.5 ± 1.7 B	11.6 ± 3.4 A	7.1 ± 2.9 a	11.4 ± 2.8 B	16.4 ± 4.5 A	5.4 ± 3.2 a
	Posterior	5.9 ± 1.5 B	11.1 ± 1.9 A	5.2 ± 1.7 b	7.8 ± 3.0 B	13.6 ± 4.0 A	5.7 ± 2.3 a

The uppercase letters represent differences between time points (paired *t* test, *p* < 0.05). The lowercase letters represent differences regarding the GBR regions (ANOVA for randomized blocks, *p* < 0.05)

## Discussion

This retrospective study demonstrates the GBR principles, using a mixture of particulate autogenous and xenogenous bone substitutes agglutinated with i-PRF, for the regeneration of horizontal and vertical bone defects. The first study describes the use of this approach for both defect types, and the data suggests that this mixture can promote good results in terms of bone gain and bring clinical and technical benefits that support their use. The concomitant use of particulate autogenous and xenogenous grafts was proposed as grafting material, a combination that already demonstrated an excellent bone gain in previous studies [25, 29]. In this mixture, the presence of autogenous bone triggers the release of osteoblasts and growth factors, while the ABBM particles promoted the volume and architecture maintenance, due to their slow reabsorption [30]. Additionally, ABBM in the mixture graft decreases the amount of autogenous bone needed, reducing the morbidity and, therefore, increasing patient comfort and satisfaction associated with these regenerative procedures [25]. In horizontal bone augmentation, the graft was protected by an absorbable collagen membrane, while in vertical defects, a non-absorbable d-PTFE-Ti membrane was preferred. The use of membranes not only protects the graft, but also promotes the isolation of the grafted region and the maintenance of space, allowing the migration of osteogenic cells to the defect, the bone remodeling, and healing [14, 31, 32]. Fixing the membranes with thumbtacks or screws stabilizes and immobilizes the region to be regenerated and seems to directly influence the final bone volume [33].

Platelet aggregates (L-PRF and i-PRF) obtained from the patients’ blood were used in this study’s protocol. Those two types of PRF were included in the treatment once they could increase the biological potential of the GBR by increasing the concentration of growth factors and other bioactive compounds that support revascularization and regeneration of hard and soft tissues [23]. Thus, it is suggested that PRF mixed with the bone graft could improve the angiogenesis, the migration of stem cells, and the osteogenic differentiation in the whole graft, favoring the graft’s integration and the clinical results [23]. In the present study, good clinical results related to the bone thickness (5.9 mm) and height (5.6 mm) gain were obtained. They are comparable to the ones obtained in horizontal (5.7 mm) and vertical (5.5 mm) gain without the use of i-PRF [25, 29], suggesting that using i-PRF in association with a bone graft can be promising. However, no clinical trial has already evaluated its real clinical benefits, and there is a lack of evidence of its benefit on clinical bone formation [17], reinforcing the need for more clinical studies in this area. Additionally to the biological improvement, the use of i-PRF to agglutinate the graft promoted a technical benefit once it facilitates the graft’s manipulation and stability [23] and improves the mechanical properties of the grafted area, which could per se support its indication.

The L-PRF is a biodegradable scaffold consisting of fibrin, platelets, and leukocytes with the potential to boost microvascularization and epithelial cell migration [34]. Therefore, they were used on top of the collagen and d-PTFE-Ti membranes in direct contact with the periosteum and soft tissues. L-PRF is related to improved soft tissue regeneration once it contains several growth factors such as platelet-derived growth factor (PDGF), transforming growth factor beta (TGF-β), vascular endothelial growth factor (VEGF), and insulin-like growth factor (IGF) [17]. Thus, their use is indicated to improve soft-tissue healing and reduce tissue dehiscence [35], reduce postoperative pain and swelling, and minimize infection in the surgical area [36]. This aspect can also be described in the present study, once no case included in this study presented post-surgery complication or membrane exposure. Reducing membrane exposure is a critical point in GBR, once it is the higher complication for this type of procedure and can critically impact the bone gain [37, 38].

For horizontal bone defects, the thickness gain was an average of 5.9 ± 2.4 mm, considering all the regenerated regions. This result is higher than the mean gain presented by other studies and systematic reviews with means of 3.61 ± 0.27 [39] and 2.2 ± 1.68 mm [33]. However, these reviews presented a higher heterogeneity in terms of surgical techniques and biomaterials. Considering a study using a similar graft mixture without agglutination with i-PRF, similar bone gain (5.68 ± 1.42 mm) was observed [25]. Besides the graft composition, it is suggested that the membrane stabilization with thumbtacks and the decorticalization of the residual bone before GBR could also interfere positively with the bone gain by increasing the grafted region’s stability and the vascularization [33]. Interestingly, the horizontal bone increase was significantly higher in the maxilla than in the mandible and in the anterior region than in the posterior region. Factors such as the bone characteristics and the architecture of the defect could directly influence the results. In the maxilla, the bone is more porous and more vascularized than the mandible, favoring the cell supply and the osteogenic property. Maxilla and in the anterior regions are usually more affected by the horizontal loss [2], which is observed by the lower baseline thickness of those regions in Table 1. More profound defects are prouder to present more significant changes after regenerative procedures than shallow defects. Furthermore, the anatomic architecture of the anterior region and the presence of teeth in both extremities of the regenerated region can also favor the graft and the membrane placement, benefiting the clinical gains and maintenance in the long term. Another factor that could explain this difference is that the patients used provisional fixed prostheses in the anterior region, as they are esthetic regions, while in posterior regions of maxillary or mandibular free extremities, no provisional prosthesis was used. It is suggested that provisional fixed prosthesis protects the grafted region, especially in cases of horizontal enlargement, treated with GBR and absorbable membrane. Despite the differences, all cases treated for the horizontal bone augmentation with GBR were able to receive the implants’ installation in the ideal tridimensional position, achieving treatment success also described by other studies [40].

The vertical augmentation promoted a height bone gain of 5.6 ± 2.6 with no difference regarding the region receiving the GBR. The observed vertical bone gain is superior to the one observed for GBR in a systematic review (4.2 mm) [41], while similar values were observed compared to a case series [29] that used a similar graft and membrane types without the addition of i-PRF for particle agglutination (5.45 ± 1.93 mm). In this situation, the use of the d-PTFE-Ti membrane was chosen to increase the occlusive capabilities and space maintenance in the regenerated area, favoring the proliferation of osteogenic cells and vascularity. In vertical defects, the angiogenic process is stimulated only from the bone in the base of the defect, reducing the cellular and vascular supply. For that reason, this type of defect is more critical to regenerate than horizontal defects [41]. The GBR with this type of membrane allows substantial bone gain with a lower complication (i.e., infection) since its porosity of fewer than 0.3 microns creates an impervious barrier to bacteria, reducing the risk and improving patient satisfaction [41]. Additionally, there was also no difference between the anterior and posterior regions and mandible and maxilla. Even though provisional prostheses were not used in posterior free extreme regions and there was of biological and anatomical differences between sites, the d-PTFE-Ti membrane could favor the bone gain and its maintenance in the long term, probably due to the higher resistance that allows better protection and higher stability of the graft.

The use of CBTC to assess bone gain obtained after GBR is a viable, reproducible, and reliable method [20, 42, 43]. The CBCT is the exam of choice for treatments with implants, once it allows the tridimensional evaluation of the residual bone, supporting the planning and the correct implant placement. Thus, the use of a CBCT after GBR and a second tomographic exam before implant placement is recommended during the dental practice. The measurement performed in the study has the premises and advantages that it used relevant anatomical points as a reference, evaluated the horizontal increases in three predetermined points along the alveolar bone crest, and measured the bone gain in the exact locations where the implants were installed. In this research, besides the tomographic examination, the grafts’ favorable result was confirmed clinically once all patients underwent surgery to install the implants in the grafted areas.

In all cases, proper incorporation of the ABBM graft and the newly formed bone crest was observed after the GBR, confirming the proposed approaches’ effectiveness. This result can be associated to many factors: the use of a mixture of the autogenous and xenogenous graft with rapid and slow reabsorption, respectively; the addition of i-PRF and growth factors to this mixture; the graft immobilization using biocompatible membranes associated with isolation of epithelial and connective cells; flap closure and first intention healing, avoiding membrane exposures and postoperative complications. Thus, the present study corroborates with the literature by demonstrating that the bone augmentation obtained with the GBR method is predictable, with fewer associated complications and stable implant installation [4, 39, 41]. However, despite the good clinical results in terms of bone gain observed with the proposed protocol, this study has the limitation of small sample size and the absence of the control group. The control group is important to evaluate the real impact of using i-PRF in association in GBR and its potential to improve bone gain. Thus, the real benefit of the used protocol, which is a mixture of autogenous and ABBM grafts with i-PRF, should still be confirmed with well-controlled randomized clinical trials and a higher sample size.

In conclusion, the results demonstrated that GBR associated with a mixture of autogenous and ABBM bone grafts agglutinated with i-PRF allowed sufficient vertical and horizontal bone augmentation, in maxillary and mandibular regions future implant placement.

## Data Availability

The datasets used and/or analyzed during the current study are available from the corresponding author on reasonable request.

## Abbreviations

GBR: Guided bone regeneration
PRF: Platelet-rich fibrin
i-PRF: Injectable form of PRF
D-PTFE-Ti: Titanium-reinforced non-resorbable high-density polytetrafluoroethylene
REBEC: Brazilian Clinical Trials Registry
CBCT: Cone-beam computed tomography
L-PRF: Leukocyte platelet-rich fibrin
ABBM: Anorganic bovine bone derived
ANOVA: Analysis of variance
PDGF: Platelet-derived growth factor
TGF-β: Transforming growth factor beta
VEGF: Vascular endothelial growth factor
IGF: Insulin-like growth factor
